# Cancer Incidence among Pesticide Applicators Exposed to Cyanazine in the Agricultural Health Study

**DOI:** 10.1289/ehp.8997

**Published:** 2006-05-31

**Authors:** Shannon M. Lynch, Jennifer A. Rusiecki, Aaron Blair, Mustafa Dosemeci, Jay Lubin, Dale Sandler, Jane A. Hoppin, Charles F. Lynch, Michael C.R. Alavanja

**Affiliations:** 1 Clinical and Genetic Epidemiology Research Branch, Epidemiology and Genetics Research Program, Division of Cancer Control and Population Sciences, National Cancer Institute, National Institutes of Health, Department of Health and Human Services, Rockville, Maryland, USA; 2 Department of Preventive Medicine and Biometrics, Uniformed Services University of the Health Sciences, Bethesda, Maryland, USA; 3 Occupational and Environmental Epidemiology Branch, Division of Cancer Epidemiology and Genetics, National Cancer Institute, National Institutes of Health, Department of Health and Human Services, Rockville, Maryland, USA; 4 Epidemiology Branch, National Institute of Environmental Health Sciences, National Institutes of Health, Department of Health and Human Services, Research Triangle Park, North Carolina, USA; 5 Department of Epidemiology, University of Iowa, Iowa City, Iowa, USA

**Keywords:** Agricultural Health Study, cancer, cyanazine, farming, herbicide, pesticides, triazine herbicide

## Abstract

**Background:**

Cyanazine is a common pesticide used frequently in the United States during the 1980s and 1990s. Animal and human studies have suggested that triazines may be carcinogenic, but results have been mixed. We evaluated cancer incidence in cyanazine-exposed pesticide applicators among the 57,311 licensed pesticide applicators in the Agricultural Health Study (AHS).

**Methods:**

We obtained detailed pesticide exposure information from a self-administered questionnaire completed at enrollment (1993–1997). Cancer incidence was followed through January 2002. Over half of cyanazine-exposed applicators had ≥ 6 years of exposure at enrollment, and approximately 85% had begun using cyanazine before the 1990s. We used adjusted Poisson regression to calculate rate ratios (RRs) and 95% confidence intervals (CIs) of multiple cancer sites among cyanazine-exposed applicators. We calculated *p*_trend_ values, and all statistical tests were two-sided. Two exposure metrics were used: tertiles of lifetime days of exposure (LD) and intensity-weighted LD.

**Results:**

A total of 20,824 cancer-free AHS applicators reported ever using cyanazine at enrollment. Cancer incidence comparisons between applicators with the lowest cyanazine exposure and those with the highest exposure yielded the following for the LD metric: all cancers, RR = 0.99 (95% CI, 0.80–1.24); prostate cancer, RR = 1.23 (95% CI, 0.87–1.70); all lymphohematopoietic cancers, RR = 0.92 (95% CI, 0.50–1.72); non-Hodgkin lymphoma, RR = 1.25 (95% CI, 0.47–3.35); lung cancer, RR = 0.52 (95% CI, 0.22–1.25).

**Conclusions:**

We did not find any clear, consistent associations between cyanazine exposure and any cancer analyzed. The number of sites was small for certain cancers, limiting any conclusion with regard to ovarian, breast, and some other cancers.

The herbicide cyanazine is a synthetic *s*-triazine (along with atrazine and simazine) that has been widely used to control broadleaf weeds and grasses in agricultural crops. It is applied as a preemergent herbicide once during the growing season to control weeds in corn, sorghum, cotton, barley, wheat, oil rape seed, sugar cane, and potatoes. Highest use of cyanazine has been in the corn-growing states of the Midwest (Snedeker and Clark 1998).

In the 1990s, cyanazine ranked as the fifth most commonly used herbicide in the United States, with an estimated 32 million pounds applied annually [U.S. Environmental Protection Agency (EPA) 2004]. Human exposure to cyanazine occurs in farming and pesticide manufacturing and through contaminated groundwater (Barbash et al. 2001; Ritter 1990) and agricultural runoff (Hansen et al. 2001). The most common application methods for cyanazine are in solution by ground boom or as a pellet, with the most common route of exposure to humans being dermal. There is little evidence to suggest that applicators are exposed to cyanazine via inhalation with recommended methods of use (U.S. EPA 1994).

The U.S. EPA classified cyanazine as a restricted use pesticide based on the detection of cyanazine in ground and surface water (i.e., restricted use pesticides may only be used by pesticide applicators certified by the state authorities), and as a Group C, possible human carcinogen based on the increased incidence of mammary tumors in rats from dietary cyanazine exposure of 25 or 50 ppm (Bogdanffy MS, unpublished data) and the possible mutagenic effect of cyanazine in mice lymphoma cells (Jannasch M, Sawin V, unpublished data). The manufacturer proposed to gradually phase out cyanazine production and use in the United States by 1999. The U.S. EPA Office of Pesticide Programs cancelled cyanazine product registrations and prohibited the sale and use of existing stocks of cyanazine after 30 September 2002 (U.S. EPA 1996). Although cyanazine is banned in the United States, it is still used in various African nations (e.g., South Africa, Niger), Asia and the Pacific Region (e.g., Australia, India, New Zealand, the Philippines), Europe (e.g., Hungary, Portugal, United Kingdom), Central Asia, Canada, and South America (Pesticide Action Network 2004).

Despite its worldwide use, studies on the health effects from cyanazine exposure specifically have been limited and results have been mixed. Studies suggest that cyanazine could be mutagenic (Jannasch M, Sawin V, unpublished data) and induce marginal DNA damage *in vivo* in mouse leukocytes administered high doses intraperitoneally (Tennant et al. 2001), but others showed no effects in human lymphocytes and rat bone marrow (Hrelia et al. 1994). Cyanazine exposure was associated with the formation of mammary-gland tumors in Sprague-Dawley rats (Bogdanffy MS, unpublished data); mechanism of action studies suggest that tumor formation is mediated through a prolactin mechanism thought to be of low relevance to the development of human breast cancer (Bogdanffy et al. 2000). However, a new study suggests that prolactin may play a larger role in the development of human breast cancer, more than previously thought (Harvey 2005).

Epidemiologic studies evaluating cancer risks associated with the triazine herbicide class and with other triazines, such as atrazine, have been conducted (Alavanja et al. 2003; Brown et al. 1990; Donna et al. 1989; Hoar et al. 1986; Hopenhayn-Rich et al. 2002; Kettles et al. 1997; MacLennan et al. 2002, 2003; Rusiecki et al. 2004; Young et al. 2004). Using a job exposure matrix to estimate cumulative exposure to triazine herbicides, Young et al. (2004) found nonstatistically significant increased odds ratios (ORs) associated with quartiles of triazine herbicide exposure and ovarian cancer [OR_high_ = 1.34; 95% confidence interval (CI), 0.42–4.28]. A case–control study found a significant increase of ovarian cancer [rate ratio (RR) = 2.7)] in triazine-exposed female farmers (Donna et al. 1989). Ecologic studies have shown a statistically significant increased risk of breast cancer with increasing triazine exposure (Kettles et al. 1997). However, another ecologic study did not find an association between atrazine exposure and breast cancer (Hopenhayn-Rich et al. 2002). In triazine-exposed manufacturing workers, greater than expected numbers of prostate, bladder, oral cavity, and lymphohematopoietic cancers were observed (MacLennan et al. 2002), but only prostate cancer was statistically significant [standardized incidence ratio (SIR) = 3.94 (95% CI, 1.28–9.20)]; all the prostate cancer cases detected were early-stage cancers, and the excess may have been due to a prostate-antigen screening program conducted at the facility (MacLennan et al. 2002). A mortality study of the same manufacturing worker population also found an increased standardized mortality ratio (SMR) for non-Hodgkin lymphoma (NHL) [SMR = 3.72 (95% CI, 1.01–9.52)] (MacLennan et al. 2003). Reported use of several individual pesticides, including atrazine, in combination with other pesticides was associated with increased NHL incidence in a case–control study in the Midwest (De Roos et al. 2003).

No association was found between atrazine exposure and prostate cancer in a study of the Agricultural Health Study (AHS) cohort (Alavanja et al. 2003). Another recent study of the AHS cohort did not find any clear association between use of atrazine and any cancer analyzed, including prostate cancer (highest exposure quartile: RR = 0.88; 95% CI, 0.63–1.23) and non-Hodgkin lymphoma (highest exposure quartile: RR = 1.61; 95% CI, 0.62–4.16) (Rusiecki et al. 2004). In case–control studies, atrazine or triazine herbicide use was also not associated with Hodgkin disease (Hoar et al. 1986), leukemia (Brown et al. 1990), multiple myeloma (Burmeister 1990), soft-tissue sarcoma (Hoar et al. 1986), or colon cancer (Hoar et al. 1985).

Given the previously high use of cyanazine in the United States, continued use in other countries, and the suggestive but incomplete data on human cancer risk, we used data from the AHS cohort to conduct the largest prospective evaluation of cyanazine exposure and cancer incidence to date. Because this cohort consisted of mostly male applicators and because the numbers of cancer cases were small for certain cancer sites of *a priori* interest, i.e., breast (*n* = 2), ovarian (*n* = 1), and oral cavity (*n* = 18), we focused this investigation on cancers for which there were at least 30 cancer cases exposed to cyanazine to ensure reasonable statistical power: prostate, all lymphohematopoietic, NHL, lung, colon, and all cancers combined.

## Methods

### Cohort enrollment and follow-up

The AHS is a prospective study of 57,311 private and commercial licensed pesticide applicators who live in Iowa and North Carolina (Alavanja et al. 1996) and were recruited between 1993 and 1997 (Alavanja et al. 1999). Members of the AHS cohort were matched to cancer registry files in both states for case identification and to state death registries and the National Death Index to ascertain vital status. Incident cancers were identified for the time period from the date of enrollment through December 2002 and were coded according to the *International Classification of Diseases for Oncology*, 2nd edition (ICD-O-2) (World Health Organization 1990). Cohort members who were alive were identified through current address records of the Internal Revenue Service (address information only), motor vehicle registration offices, and pesticide license registries of the state agricultural departments. Person-year accumulation for cancer incidence of individuals who had moved from Iowa or North Carolina was censored in the year they departed, although they were still followed up for mortality. The mean time of follow-up was 7.5 years. More than half of the applicators exposed to cyanazine had ≥ 6 years of exposure at the time of enrollment, and approximately 85% of the applicators had begun using cyanazine before 1990. All participants provided verbal informed consent, and the protocol was approved by all appropriate institutional review boards.

### Exposure assessment

Study participants were asked to complete a self-administered questionnaire at the time of enrollment, which collected comprehensive exposure data on 22 pesticides, information on ever/never use for 28 additional pesticides, use of personal protective equipment, pesticide application methods, pesticide mixing, equipment repair, lifestyle factors, cancer history, and other demographic factors. Applicators completing this questionnaire were given additional take-home questionnaires, which sought additional information on occupational exposures (commercial and private applicator questionnaire data version P1REL0310.02 and cancer registry/mortality data version AHSREL0412.01 were used in this analysis). The questionnaires may be accessed at http://www.aghealth.org/questionnaires.html (National Institutes of Health 2004).

We constructed two cumulative lifetime cyanazine exposure metrics for this analysis, each categorized into terthiles, based on the tertile levels among all cancer cases. The lifetime days of exposure (LD) calculation (years of use × number of days used per year), resulted in the following tertiles: 1–16, 17–56, and ≥ 57 lifetime days. The second exposure metric, intensity-weighted LD (IWLD) (years of use × number of days used per year × intensity level), resulted in the following tertiles: 1–83, 84–314.35, and ≥ 315.35 IWLD. To determine the number of days in an average year and the number of lifetime years, respectively, we asked each participant who indicated ever exposure to cyanazine to choose from a range of days per year and years applied. The midpoint of the indicated range for both years applied and days per year applied were used to calculate the exposure metrics. We estimated intensity levels using questionnaire data from enrollment and measurement data from the published pesticide exposure literature, as follows: intensity level = [(mixing status + application method + equipment repair status) × personal protective equipment use] (Dosemeci et al. 2002). We investigated those cancer sites for which there were at least 30 cases and 9 cases in each exposure category. We split the upper tertile at the median whenever it was possible to further investigate possible exposure–response trends.

### Statistical analysis

Prevalent cancer cases identified at or before enrollment (*n* = 1,075) and applicators who did not provide information on cyanazine use (*n* = 5,436) or were missing exposure information (*n* = 483) were excluded from this analysis, leaving 50,317 applicators. Our analyses included primary, incident cancer cases only. To examine internal exposure–response relationships, we used Poisson regression to estimate and compare RRs and 95% CIs associated with tertiles of LD (RR_ld_) and IWLD (RR_iwld_). We used two groups for reference: those reporting no use of cyanazine and those in the lowest tertile of lifetime days of use.

RRs were adjusted for age at enrollment (as a continuous variable), sex, race (white/non-white), education level [high school/general equivalency diploma (GED) or lower, beyond high school], alcohol consumption at enrollment (ever, never), family history of cancer in first-degree relatives (yes/no), state of residence (Iowa/North Carolina), and cigarette smoking history [never/low/high: median value of pack-years (12) among smokers classified low and high categories of smokers]. For each of the cumulative exposure metrics, LD and IWLD, tests for trend were carried out using the midpoints of each tertile entered into the model as a continuous variable; all statistical tests were two-sided. Potential confounding from exposure to other pesticides was controlled by adjusting for the number of days of any pesticide use and exposure to the five most highly correlated pesticides: metolachlor, alachlor, *s*-ethyl dipropylthiocarbamate (EPTC), imazethapyr, and trifluralin. These five pesticides were identified from the 50 pesticides assessed in the AHS, based on either the strength of the correlation coefficient for IWLD (highest *r* = 0.60, lowest *r* = 0.53) or the strength of association for ever/never comparisons between cyanazine and each of the 28 pesticides with ever/never data only. In the final models of the regression analyses, exposure to five highly correlated pesticides was categorized as ever/never use of the respective pesticide.

## Results

Table 1 presents the selected characteristics of the applicators in the AHS. Among the 50,800 subjects with complete exposure information, 20,341 reported ever having used cyanazine. The cohort comprised mostly white, male, private applicators with relatively low smoking rates; in both the exposed and nonexposed groups, about half had never smoked. The exposed and nonexposed groups were similar in respect to most baseline characteristics; however, the low exposed group was more similar to the higher exposed group than the nonexposed group in state of residence, corn production, and exposure to the five most highly correlated pesticides.

The Poisson regression RRs for selected cancers among cyanazine-exposed applicators, using the lowest tertile as the referent, are presented in Table 2. We split the top tertiles of exposure for all cancers, all lymphohematopoietic cancers, NHL, and prostate cancer, and results were not different (data not shown). We found no evidence of an association for all cancers combined. Prostate cancer was the most frequent cancer in the cyanazine-exposed cohort (*n* = 258). Prostate cancer showed a slight excess among the exposed for LD (RR = 1.23; 95% CI, 0.87–1.70) and IWLD (RR = 1.15; 95% CI, 0.83–1.58), and a statistically significant rate of 1.39 was observed in the medium tertile for IWLD. However, no evidence of a statistically significant exposure–response trend was seen. When subjects were stratified into those with a family history of prostate cancer and those without, there was no evidence that cyanazine use was associated with prostate cancer in either of the groups.

For all lymphohematopoietic cancers (RR = 0.92; 95% CI, 0.50–1.72) and colon cancer (RR = 0.83; 95% CI, 0.39–1.77), there were slight deficits in the highest exposure group for lifetime days. For NHL, a small, nonstatistically significant increased risk for IWLD (RR = 1.43; 95% CI, 0.61–3.37) was observed; however, we found no exposure–response association after splitting the third tertile. For lung cancer, there was a slight, nonstatistically significant decrease in estimates with increasing cyanazine use. No interaction between smoking history and lung cancer occurred in our data (data not shown).

Where nonexposed persons were used as the referent, there was a statistically significant decreased risk associated with all exposure categories for LD (highest tertile: RR = 0.84; 95% CI, 0.70–0.99) for all cancers combined. The *p*_trend_ for IWLD for all cancers (*p* = 0.02) was also statistically significant (lowest tertile: RR = 0.82; 95% CI, 0.76–1.04; medium tertile: RR = 0.88; 95% CI, 0.70–0.97; highest tertile: RR = 0.79; 95% CI, 0.67–0.93). Using the non-exposed group as the referent, we found no evidence of a statistically significant increased risk or association with cyanazine exposure for prostate, all lymphohematopoietic, NHL, colon, and lung cancers.

## Discussion

We found no clear and consistent associations between the incidence of any cancers analyzed (i.e., prostate, all lymphohematopoietic, NHL, colon, and lung cancers) using LD and IWLD as exposure metrics. Overall cancer risk patterns among exposed individuals were similar regardless of the referent group used or the exposure metric employed. In our study, we had limited power to detect a significant difference in risk for a number of cancer sites but had larger numbers to investigate the risk of prostate cancer with cyanazine exposure. Our study did not support the observed excess of prostate cancer risk in a Louisiana plant manufacturing triazine pesticides observed by MacLennan et al. (2002) [SIR = 394 (95% CI, 128–902)]. MacLennan et al. (2002) suggest this association may have occurred because prostate specific antigen screening was carried out frequently among the study cohort compared with the referent population. For prostate cancer, a nonstatistically significant increased risk among cyanazine-exposed applicators was noted when using low exposed as the referent group. However, this pattern was not observed, and the RRs were not elevated for prostate cancer when the nonexposed group was used as the referent. There was no consistent monotonic trend when the low exposed group was used the referent. This lack of consistency mitigates the possibility of an association between cyanazine and prostate cancer. A previous investigation of prostate cancer in the AHS reported by Alavanja et al. (2003) also did not find a statistically significant association between the triazine herbicide, atrazine, and prostate cancer.

We found no evidence of a statistically significant association between cyanazine exposure and all lymphohematopoietic cancers combined, NHL, or colon cancer. In a prospective cohort study of triazine herbicide manufacturing workers, MacLennan et al. (2002) found a nonsignificant increased SIR for all lymphatic and hematopoietic cancers (*n* = 7; 4.4 expected) and NHL (*n* = 3; 2.3 expected). A mortality study based on the same population found nonsignificant increased mortality ratios for NHL (*n* = 4; 1.1 expected); however, the study did not have statistical power to assess trends in rates by years worked and years since first hire (MacLennan et al. 2003). Case–control studies found no association with NHL and cyanazine exposure (De Roos et al. 2003) or between colon cancer and triazine herbicide exposure (Burmiester 1990).

For lung cancer there were nonsignificant decreased RRs with increasing tertiles of LD and IWLD for both referent groups. Because neither the risk estimates nor the tests for trend were statistically significant, and because we had no *a priori* hypothesis suggesting this trend, this may be a chance finding. Undetected residual confounding by factors such as diet, exercise, or another environmental exposure are unlikely to explain these observations, because these factors are not associated with cyanazine exposure.

Lower risks of lung cancer were seen among textile workers exposed to endotoxins in the textile dust, despite smoking habits of the workers (Levin et al. 1987). Agricultural pesticide applicators can be exposed to endotoxins from hay, grain, and animals. However, in our analyses, endotoxin exposure does not appear to account for the negative association between cyanazine exposure and lung cancer (data not shown), because there was not an association between lung cancer and any measure of farm exposure.

The AHS has several important strengths. It is the largest study to date of pesticide applicators exposed to cyanazine. Because comprehensive questionnaire data were used to quantify cyanazine exposure levels, we were able to provide greater discrimination between potential high and low exposures to cyanazine. The AHS has information on many potential cancer risk factors and can control for important confounders. It also controls for potential biases. Recall bias is minimized because exposure information was collected before cancer diagnosis. Two control groups—low exposed pesticide applicators and nonexposed applicators—were used in this study to verify study results. In general, farmers provide reliable information and considerable detail regarding their pesticide history (Blair and Zahm 1993; Blair et al. 1997, 2002; Hoppin et al. 2002). There is a lack of evidence for substantial selection bias in the AHS; the responses on the enrollment questionnaire of farmers who completed and returned the take-home questionnaire were remarkably similar to the responses on the enrollment questionnaire of farmers who did not return the take-home questionnaire (Tarone et al. 1997).

Certain limitations of our data set reduce the number and kinds of inferences we can make regarding cyanazine and its association with specific cancers. Although the AHS cohort is large and many participants reported cyanazine use, the small numbers of female applicators and the small numbers of some cancers limited our conclusions about certain cancers at this time. The average age of the applicators in our cohort is 56 years. The observational power of the AHS will increase markedly in the next few years as the cohort continues to age.

Most of the cyanazine pesticide applicators were white males (99%), limiting our ability to analyze female cancers of particular interest including breast and ovarian cancers (Bogdanffy MS, unpublished data; Donna et al. 1989; Kettles et al. 1997; Young et al. 2004). However, in a study by Engel et al. (2005), cyanazine exposure was not associated with increased breast cancer risk in wives of private applicators in the AHS who either personally used cyanazine or whose husbands used cyanazine.

Despite some limitations, our prospective study of cancer incidence among cyanazine-exposed pesticide applicators was unlike other studies, because we could evaluate cancer risks associated with exposure to cyanazine, specifically for all cancers, prostate, all lymphohematopoietic, NHL, colon, and lung cancers, while adjusting for lifestyle factors, common pesticide exposures, and other confounders. No clear, statistically significant increased risk of any of the specific cancers was observed among the 607 cyanazine-exposed cancer cases. The findings of this cyanazine study complement those found in the atrazine study (Rusiecki et al. 2004). Further detail on cancer risk, including the risk of the less frequent cancers (e.g., ovarian, breast), will be possible with continued follow-up of the AHS cohort.

## Figures and Tables

**Table 1:** Selected characteristics of applicators by cyanazine exposure in the AHS based on 1993–1997 enrollment data [no. (%)].

Characteristics	Nonexposed (*n* = 29,976)	Lowest exposed (*n* = 5,710)^a^	Highest exposed (*n* = 14,631)^b^
Age (years)			
< 40	10,693 (35.7)	1,707 (29.9)	4,468 (30.5)
40–49	7,794 (26)	1,749 (30.6)	4,787 (32.7)
50–59	5,848 (19.5)	1,219 (21.4)	3,131 (21.4)
≥ 60	5,640 (18.8)	1,035 (18.1)	2,245 (15.3)
Sex			
Male	28,814 (96.1)	5,662 (99.2)	14,537 (99.4)
Female	1,162 (3.9)	48 (0.8)	94 (0.6)
Race			
White	28,997 (96.7)	5,645 (98.9)	14,428 (98.6)
Nonwhite	895 (3)	50 (0.9)	170 (1.2)
Missing	84 (0.3)	15 (0.2)	33 (0.2)
State of residence			
Iowa	16,167 (53.9)	4,896 (85.7)	13,048 (89.2)
North Carolina	13,809 (46.1)	814 (14.3)	1,583 (10.8)
Applicator type^c^			
Private	27,279 (91)	5,491 (96.2)	12,909 (88.2)
Commercial	2,697 (9)	219 (3.8)	1,722 (11.8)
Smoking history			
Never	15,436 (51.5)	3,217 (56.3)	8,212 (56.1)
Low (< 12 pack-years)	6,399 (21.3)	1,276 (22.4)	3,268 (22.3)
High (≥ 12 pack-years)	6,916 (23.1)	1,086 (19)	2,821 (19.3)
Missing	1,225 (4.1)	131 (2.3)	330 (2.3)
Alcohol consumption			
No	10,632 (35.5)	1,446 (25.3)	3,365 (23)
Yes	18,808 (62.7)	4,210 (73.7)	11,127 (76.1)
Missing	536 (1.8)	54 (0.95)	139 (0.95)
Educational level			
≤ High school/GED	17,223 (57.5)	3,075 (53.9)	8,165 (55.8)
> High school	12,664 (42.2)	2,626 (46)	6,451 (44.1)
Missing	89 (0.3)	9 (0.16)	15 (0.1)
Family history of cancer^d^			
No	17,243 (57.5)	3,047 (53.4)	8,010 (54.7)
Yes	10,654 (35.5)	2,379 (41.7)	5,962 (40.7)
Missing	2,079 (6.9)	284 (4.9)	659 (4.5)
Corn production			
No	11,960 (39.9)	726 (12.7)	2,347 (16)
Yes	18,016 (60.1)	4,984 (87.3)	12,284 (84)
Ever exposure to five most highly correlated with cyanazine			
Metolachlor	9,736 (32.5)^e^	3,476 (60.9)^f^	9,362 (64)^g^
EPTC	2,972 (9.8)^e^	1,618 (28.3)^f^	5,412 (37)^g^
Alachlor	10,922 (36.4)^e^	3,930 (68.8)^f^	10,668 (72.9)^g^
Imazethapyr	8,600 (28.7)^e^	3,256 (57.0)^f^	8,939 (61.1)^g^
Trifluralin	10,927 (36.5)^e^	3,840 (60.9)^f^	10,516 (71.9)^g^

a First tertile of LD (years of use × days of use per year).

b Second and third tertiles of LD (years of use × days of use per year).

c Private applicators are primarily individual farmers; commercial are professional pesticide applicators.

d First-degree relatives.

e Ever exposed to indicated chemical but not to cyanazine (thus, numbers in columns do not sum to 100%).

f Ever exposed to indicated chemical and in lowest tertile of cyanazine exposure (thus, numbers in columns do not sum to 100%).

g Ever exposed to indicated chemical and in the highest two tertiles of cyanazine exposure (thus, numbers in columns do not sum to 100%).

**Table 2:** RRs (95% CIs) for selected cancers^a^ by LD and IWLD to cyanazine^b^ using low exposure as the referent.

		LD		IWLD		
Cancer site/tertile cut points^c^	No.^d^	RR (95%CI)^e^	*p*_trend_^f^	No.^d^	RR (95% CI)^g^	*p*_trend_^f^
All cancers						
1–16	174	1.00 (referent)		198	1.00 (referent)	
17–56	256	1.04 (0.86–1.27)		203	1.07 (0.88–1.30)	
≥ 57	180	0.99 (0.80–1.24)	0.79	206	0.94 (0.77–1.15)	0.35
Prostate						
1–16	67	1.00 (referent)		74	1.00 (referent)	
17–56	115	1.22 (0.89–1.65)		98	1.39 (1.03–1.88)*	
≥ 57	76	1.23 (0.87–1.70)	0.43	85	1.15 (0.83–1.58)	0.93
All lymphohematopoietic						
1–16	22	1.00 (referent)		25	1.00 (referent)	
17–56	30	0.98 (0.56–1.70)		21	0.87 (0.49–1.56)	
≥ 57	22	0.92 (0.50–1.72)	0.80	26	0.92 (0.52–1.62)	0.88
NHL						
1–16	9	1.00 (referent)		10	1.00 (referent)	
17–56	18	1.56 (0.69–3.50)		12	1.30 (0.56–3.00)	
≥ 57	9	1.25 (0.47–3.35)	0.97	13	1.43 (0.61–3.37)	0.49
Colon						
1–16	16	1.00 (referent)		20	1.00 (referent)	
17–56	16	0.69 (0.35–1.39)		13	0.69 (0.34–1.39)	
≥ 57	15	0.83 (0.39–1.77)	0.96	14	0.57 (0.27–1.17)	0.21
Lung						
1–16	15	1.00 (referent)		16	1.00 (referent)	
17–56	15	0.69 (0.33–1.44)		12	0.76 (0.36–1.63)	
≥ 57	9	0.52 (0.22–1.25)	0.25	11	0.56 (0.25–1.26)	0.12

a Cancers for which there were at least 30 exposed cases. RRs were adjusted for age, race, sex, alcohol consumption, smoking status, education level, family history of cancer, state of residence, and use of the five most highly correlated pesticides with cyanazine.

b Total number exposed to cyanazine without precancer history prior to enrollment and missing exposure information = 20,341.

c Tertiles of LD. Units for IWLD are not displayed in this table because they have intrinsic value.

d Number of cancer-specific case patients exposed to cyanazine (total and for each tertile of exposure).

e RR_ld_ = RR of LD (i.e., years of use × number of days of use per year).

f *p*-Values were two-sided.

g RR_iwld_ = RR of IWLD (i.e., years of use × number of days of use per year × intensity index).

* Indicates statistical significance.

## References

[b1-ehp0114-001248] Alavanja MC, Samanic C, Dosemeci M, Lubin J, Tarone R, Lynch CF (2003). Use of agricultural pesticides and prostate cancer risk in the Agricultural
Health Study cohort. Am J Epidemiol.

[b2-ehp0114-001248] Alavanja MC, Sandler DP, McDonnell CJ, Lynch CF, Pennybacker M, Zahm SH (1999). Characteristics of pesticide use in a pesticide applicator cohort; the
Agricultural Health Study. Environ Res.

[b3-ehp0114-001248] Alavanja MC, Sandler DP, McMaster SB, Zahm SH, McDonnell CJ, Lynch CF (1996). The Agricultural Health Study. Environ Health Perspect.

[b4-ehp0114-001248] Barbash JE, Thelin GP, Kolpin DW, Gilliom RJ (2001). Major herbicides in ground water: results from the National Water-Quality
Assessment. J Environ Qual.

[b5-ehp0114-001248] Blair A, Stewart PA, Kross B, Ogilvie L, Burmeister LF, Ward MH (1997). Comparison of two techniques to obtain information on pesticide use from
Iowa farmers by interview. J Agric Saf Health.

[b6-ehp0114-001248] Blair A, Tarone R, Sandler D, Lynch C, Rowland A, Wintersteen W (2002). The reliability of reporting on lifestyle and agricultural factors by a
sample of participants in the Agricultural Health Study from Iowa. Epidemiology.

[b7-ehp0114-001248] Blair A, Zahm SH (1993). Patterns of pesticide use among farmers: implications for epidemiological
research. Epidemiology.

[b8-ehp0114-001248] Bogdanffy MS, O’Connor JC, Hansen JF, Gaddamidi V, Van Pelt CS, Green JW (2000). Chronic toxicity and oncogenicity bioassay in rats with the chloro-*s*-triazine herbicide cyanazine. J Toxicol Environ Health.

[b9-ehp0114-001248] Brown LM, Blair A, Gibson R, Everett GD, Cantor KP, Schuman LM (1990). Pesticide exposures and other agricultural risk factors for leukemia among
men in Iowa and Minnesota. Cancer Res.

[b10-ehp0114-001248] Burmeister LF (1990). Cancer in Iowa farmers: recent results. Am J Ind Med.

[b11-ehp0114-001248] De Roos AJ, Zahm SH, Cantor KP, Weisenburger DD, Holmes FF, Burmeister LF (2003). Integrative assessment of multiple pesticides as risk factors for non-Hodgkin’s
lymphoma among men. Occup Environ Med.

[b12-ehp0114-001248] Donna A, Crosignani P, Robutti F, Betta PG, Bocca R, Mariani N (1989). Triazine herbicides and ovarian epithelial neoplasms. Scand J Work Environ Health.

[b13-ehp0114-001248] Dosemeci M, Alavanja MCR, Rowland AS, Mage D, Zahm SH, Rothman N (2002). A semi-quantitative approach for estimating exposure to pesticides in the
Agricultural Health Study. Ann Occup Hyg.

[b14-ehp0114-001248] Engel LS, Hill DA, Hoppin JA, Lubin JH, Lynch CF, Pierce J (2005). Pesticide use and breast cancer risk among farmers’ wives in the
Agricultural Health Study. Am J Epidemiol.

[b15-ehp0114-001248] Hansen NC, Moncrief JF, Gupta SC, Capel PD, Olness AE (2001). Herbicide banding and tillage system interactions on runoff losses of alachlor
and cyanazine. J Environ Qual.

[b16-ehp0114-001248] Harvey PW (2005). Human relevance of rodent prolactin-induced non-genotoxic mammary carcinogenesis: prolactin
involvement in human breast cancer and significance
for toxicology risk assessments. J Appl Toxicol.

[b17-ehp0114-001248] Hoar SK, Blair A, Holmes FF, Boysen C, Robel RJ (1985). Herbicides and colon cancer. Lancet.

[b18-ehp0114-001248] Hoar SK, Blair A, Holmes FF, Boysen CD, Robel RJ, Hoover R (1986). Agricultural herbicide use and risk of lymphoma and soft-tissue sarcoma. JAMA.

[b19-ehp0114-001248] Hopenhayn-Rich C, Stump ML, Browning SR (2002). Regional assessment of atrazine exposure and incidence of breast and ovarian
cancers in Kentucky. Arch Environ Contam Toxicol.

[b20-ehp0114-001248] Hoppin JA, Yucel F, Dosemeci M, Sandler DP (2002). Accuracy of self-reported pesticide use duration information from licensed
pesticide applicators in the Agricultural Health Study. J Expo Anal Environ Epidemiol.

[b21-ehp0114-001248] Hrelia P, Vigagni F, Maffei F, Morotti M, Colacci A, Perocco P (1994). Genetic safety evaluation of pesticides in different short-term tests. Mutat Res.

[b22-ehp0114-001248] Kettles MK, Browning SR, Prince TS, Horstman SW (1997). Triazine herbicide exposure and breast cancer incidence: an ecologic study
of Kentucky counties. Environ Health Perspect.

[b23-ehp0114-001248] Levin LI, Gao YT, Blot WJ, Zheng W, Fraumeni JF (1987). Decreased risk of lung cancer in the cotton textile industry of Shanghai. Cancer Res.

[b24-ehp0114-001248] MacLennan PA, Delzell E, Sathiakumar N, Myers SL (2003). Mortality among triazine herbicide manufacturing workers. J Toxicol Environ Health.

[b25-ehp0114-001248] MacLennan PA, Delzell E, Sathiakumar N, Myers SL, Cheng H, Grizzle W (2002). Cancer incidence among triazine herbicide manufacturing workers. J Occup Environ Med.

[b26-ehp0114-001248] National Institutes of Health 2004. Agricultural Health Study Homepage. Bethesda, MD:National Institutes of Health. Available: http://www.aghealth.org [accessed 25 September 2004].

[b27-ehp0114-001248] Pesticide Action Network 2004. PAN Pesticides Database—Pesticide Registration Status. Available: http://www.pesticideinfo.org/Detail_ChemReg.jsp?Rec_Id=PC33516 [accessed 1 October 2004].

[b28-ehp0114-001248] Ritter WF (1990). Pesticide contamination of ground water in the United States—a
review. J Environ Sci.

[b29-ehp0114-001248] Rusiecki JA, De Roos A, Lee WJ, Dosemeci M, Lubin JH, Hoppin JA (2004). Cancer incidence among pesticide applicators exposed to atrazine in the
Agricultural Health Study. J Natl Cancer Inst.

[b30-ehp0114-001248] Snedeker SM Clark H 1998. Critical evaluation of cyanazine’s breast cancer risk. Program on Breast Cancer and Environmental Risk Factors in New York State (BCERF). Available: http://envirocancer.cornell.edu/FactSheet/Pesticide/fs17.cyanazine.cfm [accessed 1 January 2005].

[b31-ehp0114-001248] Tarone RE, Alavanja MCR, Zahm SH, Lubin JH, Sandler DP, McMaster MP (1997). The Agricultural Health Study: factors affecting completion and return
of self-administered questionnaires in a large prospective cohort study
of pesticide applicators. Am J Ind Med.

[b32-ehp0114-001248] Tennant AH, Peng B, Kligerman AD (2001). Genotoxicity studies of three triazine herbicides: *in vivo* studies using the alkaline single gel (SCG) assay. Mutat Res.

[b33-ehp0114-001248] U.S. EPA (U.S. Environmental Protection Agency) 1994 Atrazine, simazine and cyanazine: notice of initiation of special review Fed Reg 59 60412 60443 Available: http://www.epa.gov/fedrgstr/EPA-PEST/1994/November/Day-23/pr-54.html [accessed 18 October 2004].

[b34-ehp0114-001248] U.S. EPA (U.S. Environmental Protection Agency) (1996). Cyanazine: notice of final determination of terminate special review of
cyanazine; notice of voluntary cancellation and cancellation order of
cyanazine product registrations. Fed Reg.

[b35-ehp0114-001248] U.S. EPA (U.S. Environmental Protection Agency) 2004. Federal Insecticide, Fungicide, and Rodenticide Act (FIFRA). Available: http://www.epa.gov/opp00001/regulating/fifra.pdf [accessed 2 January 2005].

[b36-ehp0114-001248] World Health Organization 1990. International Statistical Classification of Disease for Oncology. 2nd ed. Geneva:World Health Organization.

[b37-ehp0114-001248] Young HA, Mill PK, Riordan R, Cress R (2004). Use of a crop and job specific exposure matrix for estimating cumulative
exposure to triazine herbicides among females in a case-control study
in the Central Valley of California. Occup Environ Med.

